# Dietary Soybean and *Kappaphycus* sp. Differentially Modulate Gut Microbiota Diversity and Predicted Functional Profile in Nile Tilapia (*Oreochromis niloticus*)

**DOI:** 10.1002/mbo3.70442

**Published:** 2026-10-18

**Authors:** Leonildo dos Anjo Viagem, Jean Nepomuscene Hakizimana, Cyrus Rumisha, Brunno da Silva Cerozi, Gerald Misinzo

**Affiliations:** ^1^ Department of Animal, Aquaculture and Range Sciences, College of Agriculture Sokoine University of Agriculture Morogoro Tanzania; ^2^ SACIDS Africa Centre of Excellence for Infectious Diseases, SACIDS Foundation for One Health Sokoine University of Agriculture Morogoro Tanzania; ^3^ Institute of Natural Resources and Environment Rovuma University Cabo Delgado Mozambique; ^4^ Department of Animal Science, College of Agriculture University of São Paulo São Paulo Brazil; ^5^ Department of Microbiology, Parasitology and Biotechnology, College of Veterinary Medicine and Biomedical Sciences Sokoine University of Agriculture Morogoro Tanzania

**Keywords:** aquaculture nutrition, functional prediction, gut microbiota, Kappaphycus sp, Nile tilapia, soybean meal

## Abstract

Diet composition shapes gut microbiota, influencing nutrient utilization, health, and growth. The shift toward sustainable ingredients such as soybean meal (SB) and seaweed (SW) requires an understanding of their effects on the gut microbiota. This study investigated how SB and SW diets modulate gut microbiota and predicted functional profile in Nile tilapia. Fish were assigned to four dietary treatments for 62 days: fishmeal (FM), SB, SW, and a natural meal (NM, non‐formulated diet). Gut microbiota were characterized using 16S rRNA gene sequencing, and functional potential was inferred using PICRUSt2. Diet significantly influenced microbial beta diversity (PERMANOVA, *R* ^2^ = 0.60, *F* = 4.05, *p* = 0.001). Alpha diversity was highest in the SW and lowest in the NM, with a significant difference detected only between these groups. *Fusobacteriota* and *Pseudomonadota* predominated across treatments, while *Cetobacterium* was consistently identified as a core genus. SB and SW diets were associated with distinct taxonomic and predicted functional profiles. SB diet showed higher representation of pathways related to nucleotide turnover, central carbon metabolism, and stress response, and was associated with growth performance. SW showed higher predicted abundances of amino acid biosynthesis and carbohydrate metabolism pathways, and achieved the highest protein digestibility. In contrast, NM showed lower predicted functional potential and lower growth indices. Overall, SB and SW were associated with changes in microbial composition and predicted metabolic functions while maintaining relatively stable core microbiota. These findings support the relevance of gut microbiota as a biological indicator when evaluating sustainable feed ingredients for Nile tilapia aquaculture.

## Graphical abstract

Soybean meal and *Kappaphycus* sp. diets reshaped gut microbiota diversity, taxonomic composition, and predicted metabolic functions in Nile tilapia while maintaining a stable core microbiota. These findings highlight gut microbial responses as a useful indicator for evaluating sustainable alternative feed ingredients in aquaculture.

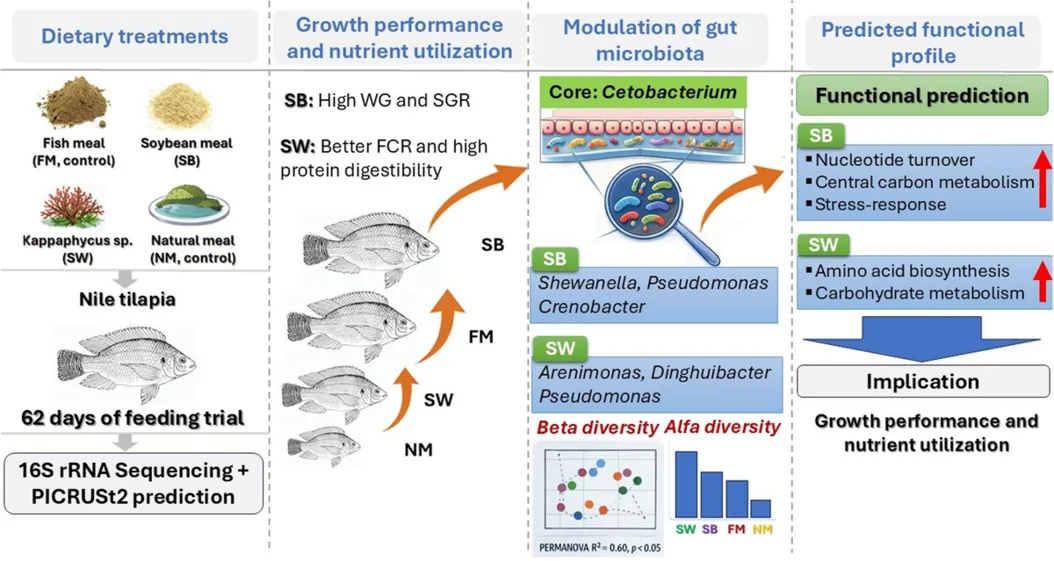

## 1 Introduction

Nile tilapia (*Oreochromis niloticus*) is one of the most widely farmed freshwater fish worldwide. It is valued for its rapid growth and broad environmental tolerance. As tilapia aquaculture becomes increasingly intensive, diet formulation has become a key factor influencing production efficiency, fish health, and environmental sustainability (Emam et al. 2025; Mendes et al. 2024). Therefore, understanding how feed ingredients affect the intestinal microbiota of *O. niloticus* is essential. The gut microbiota plays important roles in nutrient digestion, vitamin synthesis, immune modulation, and pathogen resistance. It also responds rapidly to changes in dietary composition and processing (Acosta et al. 2022; Talwar et al. 2018). Studies of tilapia gut microbiota have identified a core but dynamic microbial community. Its composition varies with life stage, rearing environment, and diet, highlighting opportunities to steer microbial function through targeted feed strategies (Bereded et al. 2020; Huang et al. 2020).

The replacement of fishmeal with plant‐based ingredients, primarily soybean meal, has been driven by economic and sustainability concerns. Soybean meal is a high‐protein and cost‐effective alternative (Akter et al. 2024; Hussain et al. 2024). However, it contains several anti‐nutritional factors, including saponins, protease inhibitors, and non‐starch polysaccharides. These compounds can affect intestinal structure and alter microbial balance, especially at high inclusion levels or when soybean meal is inadequately processed (Gopan et al. 2020; Sinha et al. 2011). Studies have shown that soybean‐based diets can modify the composition and metabolic activity of the fish gut microbiota. They may also reduce mucosal barrier integrity and contribute to dysbiosis in some cases (Miao et al. 2018; Solis et al. 2020). However, strategies such as enzyme supplementation and improved processing can reduce these adverse effects. They may also promote microbial communities associated with beneficial functions (Wang et al. 2023).

Seaweed, particularly red seaweeds such as *Kappaphycus* spp., is receiving increasing attention as a multifunctional feed ingredient. *Kappaphycus* is rich in sulfated polysaccharides (*k*‐carrageenans), pigments, micronutrients, and diverse secondary metabolites. These compounds can act as prebiotic substrates, immunostimulants, and feed binders (Guillén et al. 2022; Hardjani et al. 2017; Situmorang et al. 2021). Studies in aquaculture species have shown that dietary seaweed or its extracts can improve growth and modulate immune responses. They also alter gut microbial communities toward taxa associated with improved barrier function and pathogen resistance. These effects are consistent with known mechanisms of seaweed polysaccharides. Although they resist digestion by the host, specific gut bacteria can ferment them and produce metabolites such as short‐chain fatty acids. These metabolites may support mucosal health and immune homeostasis (Siddik et al. 2023).

Despite growing interest in soybean and seaweed ingredients, direct comparisons of their effects on tilapia gut microbial diversity and functional capacity remain limited. This study investigated how soybean and a diet containing red seaweed (*Kappaphycus* sp.) affect the diversity and functional profile of the gut microbiota in Nile tilapia (*Oreochromis niloticus*). We hypothesized that replacing fishmeal with soybean meal or *Kappaphycus* sp. would produce distinct changes in microbial community structure and predicted metabolic functions. These changes may reflect differences in nutrient utilization and ultimately affect fish growth performance.

## 2 Materials and Methods

### 2.1 Diet Formulation

Four experimental diets were prepared as described in our previous study (Viagem et al. 2026). Briefly, three formulated diets were used: a fishmeal‐based diet (FM, positive control), a soybean meal diet (SB), and a red seaweed diet containing *Kappaphycus* sp. (SW). The fourth experimental diet was a natural meal (NM), in which fish received no formulated feed, and served as the negative control.

The NM treatment was not intended to represent a nutritionally equivalent diet but rather an ecological reference representing fish that relied exclusively on naturally available food resources within the culture environment. This treatment was included to distinguish microbiota characteristics associated with natural environmental feeding from those associated with formulated diets. Because nutrient intake in this group is neither quantified nor controlled, comparisons with formulated diets should be interpreted as contrasts between environment‐driven and diet‐driven microbial assembly rather than direct nutritional equivalence.

All diets were formulated using WinFeed 2.8 software and designed to be isoproteic (36% crude protein) and isoenergetic (3600 kcal/kg) (Table 1). Fishmeal (Patense Ltda., Patos de Minas, Minas Gerais, Brazil) and toasted soybean meal (Caramuru Alimentos S/A, Itumbiara, Brazil) were obtained from local suppliers, while *Kappaphycus* sp. was provided by ALGATECH (Ubatuba, Brazil). All ingredients were ground, weighed using a precision balance (FSH‐5000, Digiscale, Germany), and thoroughly mixed. The mixture was extruded at 80°C using an Inbramaq Mx‐30 extruder (Inbramaq, Ribeirão Preto, Brazil), formed into 3‐mm pellets, and dried at 45°C for 24 h. The final diets were stored in plastic bags until use.

**Table 1:** Ingredients and proximate composition analysis of experimental diets.

Ingredient (%)	Diets			
	FM	NM	SB	SW
Fish meal	40	—	—	—
Soybean^a^	—	—	42	—
Seaweed	—	—	—	10
Wheat gluten	10	—	15	35
Maize bran	24	—	23	30
Rice bran	25	—	17	22
Maize oil	—	—	2	2
Vitamin and mineral Premix^b^	1	—	1	1
**Proximate composition**				
Dry matter (g/kg)	940.48	—	938.68	929.99
Crude protein (g/kg)	364.8	—	364.0	363.0
Crude fiber (g/kg)	30.4	—	40.6	45.1
Crude fat (g/kg)	4.07	—	3.79	3.72
Ash (g/kg)	94.0	—	55.1	70.1
Carbohydrate (g/kg)	204.9	—	258.9	405
Tannin (g/kg)	2.04	—	2.45	3.72
Energy (kcal/kg)	3639	—	3637	3635

Abbreviations: FM: fish meal (positive control); NM: natural meal (negative control) was not a formulated experimental feed, but rather a natural meal used solely as a baseline reference; SB: soybean meal diet; SW: diet containing seaweed (*Kappaphycus* sp., crude protein: 84.9 g/kg).

a Toasted soybean.

b 1 kg of mixture contains folic acid: 250 mg; pantothenic acid: 5000 mg; mixed herb flavoring: 50 mg; BHT: 15 g; biotin: 50 mg; copper: 1995 mg; choline: 50 g; onion extract: 87 mg; grape seed extract: 15.6 mg; Iron: 13. 47 g; iodine: 75 mg; manganese: 3733 mg; niacin: 5000 mg; calcium propionate: 200 mg; selenium: 75 mg; vitamin A: 1000000UI; vitamin B1: 500 mg; vitamin B12: 3750 mg; vitamin B2: 1750 mg; vitamin B6: 2488 mg; vitamin C: 25 g; vitamin D3: 500000UI; vitamin E: 18750UI; vitamin K3: 500 mg; zinc: 20 g.

Proximate composition analysis of the experimental diets (Table 1) was conducted at the Animal Nutrition Laboratory (CENA, Piracicaba, Brazil) using AOAC (2005) standard procedures as described in our previous study (Viagem et al. 2026). Moisture content was determined by oven drying at 105°C for 24 h, while crude protein was measured via the Kjeldahl method (Nitrogen × 6.25). Fat content was assessed through Soxhlet extraction, and ash content by incineration at 550°C for 6 h. Crude fiber was analyzed using acid‐base digestion. Carbohydrates were quantified by a colorimetric method (Dubois et al. 1956), and tannin levels were measured using the Folin‐Denis procedure (Makkar 2003).

### 2.2 Fish Rearing

The feeding trial was conducted as described in our previous study (Viagem et al. 2026). Briefly, reverse‐sex Nile tilapia (*Oreochromis niloticus*) fingerlings were obtained from a local commercial hatchery. Upon arrival, fish were stocked in 1000 L fiberglass tanks operating under a continuous open‐flow water system and maintained for 4 weeks. Afterward, fish averaging 6.37 ± 0.60 g were acclimated to experimental conditions for 15 days while fed a basal diet. At the end of acclimation, fish averaging 9.98 ± 2.12 g were randomly distributed in four dietary treatments, each conducted in triplicate. The experiment was conducted in 12 rectangular plastic tanks (300 L capacity) equipped with an open‐flow system. Stocking density was set at 20 fish per tank, corresponding to 60 fish per treatment. The feeding trial lasted 62 days. Fish were fed daily at 3% of their total biomass, with the ration divided into three equal meals. Every 2 weeks, fish were bulk‐weighed to assess growth performance and adjust feed allowances accordingly. Daily feed intake was recorded, and uneaten feed was collected 30 min after each meal, dried to a constant weight, and deducted from the total amount offered to accurately estimate feed consumption.

Water quality variables were monitored throughout the experimental period to ensure comparable environmental conditions among treatments. Water temperature was maintained at 28°C using submersible heaters, and dissolved oxygen (6.89 ± 0.20 mg/L) was monitored daily, whereas pH (7.98 ± 0.10) and total ammonia (0.01 ± 0.00 µmol/L) were measured weekly. These parameters remained within acceptable ranges for Nile tilapia culture and showed no significant differences among treatments. A 12 h light:dark photoperiod was maintained throughout the experiment, and tanks were cleaned regularly to remove accumulated waste while minimizing disturbance to the fish. For the negative control (NM) treatment, continuous water flow was intentionally avoided to preserve natural productivity (periphyton and naturally occurring particulate matter) and prevent the loss of dietary particulate material. Instead, water was renewed manually twice per week, maintaining water quality while minimizing feed washout and ensuring consistent dietary exposure under comparable environmental conditions.

### 2.3 Determination of Growth Performance, Feed Utilization, and Nutrient Digestibility

The weight gain (WG), specific growth rate (SGR), condition factor (CF), survival ratio (SR), feed intake (FI), and feed conversion ratio (FCR) were calculated as follows:

$$\mathrm{SGR}\;\left(\%/\mathrm{day}\right)=\frac{\left[\mathrm{Ln}(\mathrm{Final}\;\mathrm{body}\;\mathrm{weight})-\mathrm{Ln}(\mathrm{Initial}\;\mathrm{body}\;\mathrm{weight})\right]}{\mathrm{Duration}\;\mathrm{of}\;\mathrm{the}\;\mathrm{experiemnt}\;\left(\mathrm{days}\right)}\;\times \;100$$

WG=Finalbodyweight(g)−Initialbodyweight(g)

$$\mathrm{FI}\;\left(g/\mathrm{fish}\right)=\frac{\mathrm{Total}\;\mathrm{feed}\;\mathrm{fed}\;(g)}{\mathrm{Number}\;\mathrm{of}\;\mathrm{fish}\;\mathrm{at}\;\mathrm{the}\;\mathrm{end}\;\mathrm{of}\;\mathrm{the}\;\mathrm{experiemnt}}$$

$$\mathrm{FCR}=\frac{\mathrm{Feed}\;\mathrm{intake}\;(g/\mathrm{fish})}{\mathrm{Weight}\;\mathrm{gain}\;(g/\mathrm{fish})}$$

$$\mathrm{SR}\;\left(\%\right)=\frac{\mathrm{Number}\;\mathrm{of}\;\mathrm{fish}\;\mathrm{at}\;\mathrm{end}\;\mathrm{of}\;\mathrm{the}\;\mathrm{experiment}}{\mathrm{Number}\;\mathrm{of}\;\mathrm{fish}\;\mathrm{at}\;\mathrm{begning}\;\mathrm{of}\;\mathrm{the}\;\mathrm{experiment}}\;\times \;100$$

$$\mathrm{CF}\;\left(\%\right)=\left[\frac{\mathrm{Body}\;\mathrm{weight}\;\left(g\right)}{{\left(\mathrm{Body}\;\mathrm{length}\;\left(\mathrm{cm}\right)\right)}^{3}}\right]\times \;100$$

The nutrient digestibility was conducted as described in our previous study (Viagem et al. 2026). Briefly, after 62 days of the feeding trial, 25 homogeneous fish (30.55 ± 0.52 g) from each diet group were selected and placed in circular fiber tanks with a capacity of 500 L each. The fish were fed 3% of their biomass twice daily over 30 days. At the end of each trial day, the fish from each group were transferred to the digestibility chamber for feces collection. Feces were collected daily before the morning feeding using collection columns immersed in an ice slurry installed below each digestibility chamber. The fecal samples were dried and pooled at 10‐day intervals, and apparent digestibility coefficients (ADCs) for dry matter, protein, fat, carbohydrate, and energy were calculated using proximate analyses of feed and feces, following the direct method described by Obirikorang et al. (2020), who have previously applied this method in tilapia nutrition studies when inert markers are not available. The formula used to calculate the ADCs was as follows:

$$\mathrm{ADC}\;\left(\%\right)=\left(\frac{\mathrm{Nutrient}\;\mathrm{in}\;\mathrm{feed}-\mathrm{Nutrient}\;\mathrm{in}\;\mathrm{faces}}{\mathrm{Nutrient}\;\mathrm{in}\;\mathrm{feed}}\right)\times \;100$$

### 2.4 Sample Collection

The fish were fasted for 24 h. Following this period, four fish from each tank (a total of twelve fish per feeding treatment) were randomly selected, anesthetized with benzocaine (132 mg/L), and humanely euthanized. Dissections were performed on an ice tray using sterilized equipment, and each intestinal sample was immediately rinsed in sterile phosphate‐buffered saline (PBS) before being transferred to a sterilized 1.5 mL Eppendorf tube. Samples were kept on ice and promptly transported to the Animal Biotechnology Laboratory at ESALQ‐USP (Piracicaba, SP, Brazil), where they were stored at −80°C until DNA extraction.

### 2.5 DNA Extraction and Quantification

Samples were individually macerated in liquid nitrogen, and the resulting material was transferred to 5‐mL tubes. Using a spatula, 50–55 mg of each macerated sample was weighed and transferred into tubes containing glass beads. Subsequently, 1 mL of InhibitEX Buffer (QIAamp Fast DNA Stool Mini Kit, Qiagen, Hilden, Germany) was added to each tube. Samples were homogenized in a FastPrep instrument at 4.5 m/s for two 1‐min cycles. DNA extraction was then performed according to the manufacturer's protocol (QIAamp Fast DNA Stool Mini Kit, Qiagen, Hilden, Germany). After DNA extraction, individual subsamples were pooled by cultivation tank to generate a single composite sample per tank. The tank was the experimental unit, and pooling was performed to reduce pseudo‐replication and emphasize treatment‐level effects. For each treatment, three pooled samples (one per tank; triplicates) were analyzed, representing tank‐level microbiota rather than individual organism‐level microbiota. The concentration and purity of the extracted DNA were measured using a NanoDrop spectrophotometer (Biochrom, Cambridge, England). Measurements were conducted with 1 µL of each sample, yielding DNA concentrations in ng/µL and purity ratios (A260/A280 and A260/A230).

### 2.6 16S rRNA Gene Amplification and Sequencing

A nested polymerase chain reaction (PCR) approach was employed to enhance the specificity and sensitivity of 16S rRNA gene detection. The first‐round PCR targeted the nearly full‐length 16S rRNA gene using universal primers (10 µM): 27 F (5′‐AGAGTTTGATCCCTCAG‐3′) and 1492 R (5′‐GGTTACCTTGTTACGACTT‐3′) (Lane 1991; Turner et al. 1999). Each 25 µL reaction contained 12.5 µL of 2× PCR master mix, 0.5 µM of each primer, 1 µL of template DNA, and nuclease‐free water. Thermal cycling conditions were initial denaturation at 95°C for 5 min; 30 cycles of 95°C for 30 s, 55°C for 30 s, 72°C for 90 s; final extension at 72°C for 7 min. The products of this amplification were visualized on 1% agarose gel electrophoresis using a UV transilluminator (Bio‐Rad Gel Doc XR+ Imaging System). After visualization, the PCR products were purified using AMPure XP Beads (Beckman Coulter, Life Sciences). The product of this reaction was used as a template for the second amplification round (nested PCR).

The second‐round (nested) PCR used 1 µL of the first‐round product as template and primers targeting the V3–V4 hypervariable regions (341 F: 5′‐CCTAYGGGRBGCASCAG‐3′, 806 R: 5′‐GGACTACNNGGGTATCTAAT‐3′) (Sundberg et al. 2013). The reaction mixture composition was identical to that of the first‐round PCR. Cycling conditions were initial denaturation at 95°C for 3 min; 25 cycles of 95°C for 30 s, 58°C for 30 s, 72°C for 45 s; final extension at 72°C for 5 min. Nested PCR products were confirmed by electrophoresis on a 1.5% agarose gel. Amplified products were purified using AMPure XP Beads (*Beckman Coulter, Life Sciences, Indianapolis, United States*). The purified nested PCR products were ligated to Illumina adapters: Nextera XT Index Primer 1 (N7xx) and Nextera XT Index Primer 2 (S5xx) index. The PCR products obtained after adapter ligation were quantified using a NanoDrop spectrophotometer and then normalized to the same concentration. An equimolar pool of all samples was prepared, which was subsequently quantified by quantitative qPCR to validate and determine the final concentration (in nM). The KAPA Library Quantification Kit for Illumina (Roche, Cat. No. 07960204001) was used for this step. Sequencing was performed on an Illumina NextSeq. 2000 platform using 2 × 300 bp runs. Libraries were prepared using the Illumina DNA Prep Kit (Catalog No. 20060059, Illumina, San Diego, CA, USA) according to the manufacturer's instructions. The resulting paired‐end libraries were sequenced on an Illumina NextSeq. 2000 platform using a 2× 300‐bp read configuration and the NextSeq. 1000/2000 P1 XLEAP‐SBS Reagent Kit (600 cycles; Cat. No. 20100981; Illumina, USA).

### 2.7 Raw‐Read Quality Control and Trimming

Raw multiplexed sequencing reads were first demultiplexed by sample, followed by an initial per‐sample quality assessment using FastQC v0.12.1 and MultiQC v1.15 to identify low‐quality cycles, adapter or primer contamination, and overall run performance. Primer and adapter sequences were then removed using Cutadapt v4.5, and the reads were imported into R (v4.4.0) for inspection with ShortRead v1.58.0 and Biostrings v2.66.0.

The quality control and processing of paired‐end amplicon sequences were conducted using the DADA2 package version 1.26 (Callahan et al. 2016), implemented in R, following Bioconductor best practices. The *filterAndTrim* function was applied to remove low‐quality bases based on per‐base quality profiles, with maxEE and truncQ parameters optimized per run, while maintaining sufficient overlap between forward and reverse reads for accurate merging. Reads containing ambiguous bases (“N”) or below minimum length thresholds were discarded. The *dada* function was then used to model amplicon errors and infer exact Amplicon Sequence Variants (ASVs), thereby avoiding traditional Operational Taxonomic Unit (OTU) clustering. Periodic in *silico* contamination assessment was conducted using Decontam v1.20.0 to identify unexpected taxa or batch‐associated contaminants.

### 2.8 Error Modeling and Denoising

DADA2 employs a parametric error model to distinguish sequencing errors from true biological variation. Error rates were estimated for each sequencing run using the learnErrors function, which iteratively alternates between error rate estimation and sample inference until convergence is achieved. The initial model assumes maximal error rates, where only the most abundant sequence is considered correct, and all others are treated as potential errors. Following error rate learning, dereplication was performed using the derepFastq function to collapse identical reads into unique sequences with corresponding abundance counts. This process also generated consensus positional quality scores by averaging base qualities across identical reads. The denoising step was then applied using the dada function to infer high‐resolution ASVs for each sample.

### 2.9 Merging of Paired‐End Reads and Chimera Removal

Unlike many OTU‐based workflows, DADA2 merges paired‐end reads after denoising, as this improves accuracy by eliminating most substitution errors before merging. The merging step was performed using the mergePairs function, which requires exact overlap with no mismatches between forward and reverse reads. Successfully merged reads were then subjected to chimera detection and removal using the removeChimeras function (method = “consensus”) to eliminate artificial sequences formed during PCR amplification. High‐quality reads that passed all filtering steps were subsequently used for downstream ASV‐based microbiota analysis.

### 2.10 Taxonomic Assignment and Microbiota Data Processing

Taxonomic classification of ASVs was performed using DADA2's implementation of the naive Bayesian classifier (assignTaxonomy function). The SILVA reference database version 138.2 was used as the training dataset for taxonomic assignment at the genus level. Taxonomic identities were recorded together with ASV abundance data for subsequent community analysis. The resulting ASV abundance table, taxonomic classifications, and sample metadata were imported into the phyloseq package (McMurdie and Holmes 2013) in R for ecological and statistical analyses. Phyloseq enables the integration, manipulation, and visualization of high‐throughput microbiome data. Community composition and diversity metrics were calculated, and relative abundance plots were generated using the ggplot2 package.

Further ecological analyses were conducted using integrated R packages such as VEGAN (for diversity and ordination analyses) (Dixon 2003), ade4 (Dray and Dufour 2007), ape (Paradis and Schliep 2019), and Picante (Kembel et al. 2010), providing a robust statistical framework for interpreting microbial community structure and phylogenetic diversity.

### 2.11 Differential Abundance of Intestinal Microbiota

The differential abundance of the intestinal microbiota was analyzed in R (version 4.4.0) using the DESeq. 2 package to identify bacterial taxa significantly affected by diet. Raw 16S rRNA sequencing counts were imported and filtered to exclude low‐abundance taxa (< 10 total counts). Data were normalized using DESeq. 2′s median‐of‐ratios method to account for differences in library size. Differential abundance between experimental groups was assessed with the Wald test, and p‐values were adjusted for multiple comparisons using the Benjamini‐Hochberg method (FDR < 0.05). Results were visualized with volcano plots.

### 2.12 Functional Profile Prediction

The functional profile of the gut microbiota was predicted using Phylogenetic Investigation of Communities by Reconstruction of Unobserved States (PICRUSt2) version 2.6.2 (Douglas et al. 2020). Amplicon sequence variants (ASVs) obtained from the DADA2 pipeline were phylogenetically placed using EPA‐ng (Barbera et al. 2019), aligning sequences within a reference tree. Gene content per ASV was inferred through hidden state prediction via the castor package (Louca and Doebeli 2018), leveraging reference genomes to estimate functional profiles. Predicted gene families were mapped to metabolic pathways using MinPath (Ye and Doak 2009), minimizing redundancy and emphasizing essential pathways. Functional profiles were generated across Enzyme Commission (EC) numbers and MetaCyc pathways (Caspi et al. 2018; Karp 2002), enabling comprehensive characterization of microbial metabolic potential. Outputs were normalized for 16S rRNA gene copy number to allow accurate cross‐sample comparisons, providing detailed insights into the putative functional capabilities of microbial communities and their potential contributions to the Nile tilapia. The metabolic pathways predicted from 16S rRNA gene sequences using PICRUSt2 were visualized in R version 4.4.0.

### 2.13 Statistical Analysis

Growth performance, feed efficiency, and nutrient digestibility were calculated using standard equations and analyzed by one‐way ANOVA to compare dietary treatments. Assumptions of normality and homogeneity of variances were evaluated using the Shapiro–Wilk and Levene's tests, respectively. When significant treatment effects were detected (*p* < 0.05), Tukey's HSD test was used for pairwise comparisons. All analyses were conducted in RStudio version 4.4.0, and results are presented as means ± SE. The experimental tank (*n* = 3 per dietary treatment) was considered the experimental unit for all analyses.

For microbiota analysis, individual fish from the same tank were pooled before DNA analysis to avoid pseudoreplication. Consequently, each tank‐derived pooled sample was treated as an independent experimental unit, and all subsequent statistical analyses were conducted at tank level. Alpha diversity was assessed using the Shannon and Simpson diversity indices. Differences in alpha diversity among dietary treatments were evaluated by one‐way ANOVA followed by Tukey's HSD test, and differences were considered significant at *p* < 0.05.

Microbial community structure was explored using detrended correspondence analysis (DCA) and multidimensional scaling (MDS). DCA was used to visualize patterns of variation in community composition, whereas MDS was performed using weighted UniFrac distances (Lozupone and Knight 2005), which incorporate phylogenetic relationships and relative taxon abundance. Differences in community composition among dietary treatments were evaluated using permutational multivariate analysis of variance (PERMANOVA) with 4,999 permutations, using the adonis2 function in the VEGAN package. Bray–Curtis dissimilarities were calculated from Hellinger‐transformed abundance data, with dietary treatment specified as the explanatory factor. Because each tank contributed a single pooled sample, permutations were unrestricted. Homogeneity of multivariate dispersions was assessed using the betadisper function followed by permutation testing. Venn diagrams were generated using Venny 2.1.0 (https://bioinfogp.cnb.csic.es/tools/venny/) to identify taxa shared among or unique to the different dietary treatments.

## 3 Results

### 3.1 Growth Performance and Nutrient Utilization

In our previous study, Viagem et al. (2026), we reported that the growth performance and feed utilization were significantly influenced by dietary treatment, whereas survival was not affected (Table 2). Initial body weight (IBW) did not differ significantly among treatments (*p* > 0.05), confirming that the treatments were comparable at the start of the experiment. In contrast, final body weight (FBW) and weight gain (WG) were significantly affected by diet (*p* < 0.05). Fish fed the soybean meal (SB) diet achieved the highest FBW and WG, followed closely by those fed diets containing fishmeal (FM) and *Kappaphycus* sp. (SW), while fish in the natural meal (NM) group exhibited lower growth performance. Specific growth rate (SGR) showed a similar pattern, with the diets containing SB, FM, and SW supporting significantly higher growth rates than the NM diet. The condition factor (CF) was significantly lower in the NM group, whereas the FM, SB, and SW diets maintained similar and higher values. Feed intake (FI), feed conversion ratio (FCR), and survival rate (SR) did not differ significantly among treatments (*p* > 0.05), indicating similar feed efficiency and survival across diets despite differences in growth performance.

**Table 2:** Growth performance and feed utilization parameters of fish fed different experimental diets.

Parameters	Experimental diet				*p *value
	FM	NM	SB	SW	
IBW (g)	9.96 ± 0.19	9.84 ± 0.27	10.05 ± 0.29	10.08 ± 0.25	0.65
FBW (g)	38.32 ± 0.83^ab^	22.15 ± 0.21^c^	39.42 ± 0.61^a^	37.71 ± 0.32^b^	< 0.001
WG (g)	28.36 ± 0.84^ab^	12.31 ± 0.14^c^	29.37 ± 0.57^a^	27.63 ± 0.45^b^	< 0.001
FI (g/fish)	30.74 ± 0.00		31.35 ± 6.19	24.85 ± 0.04	0.12
SGR (%)	2.17 ± 0.04^a^	1.31 ± 0.03^b^	2.20 ± 0.04^a^	2.13 ± 0.05^a^	< 0.001
FCR	1.08 ± 0.03		1.07 ± 0.21	0.90 ± 0.02	0.21
CF (%)	2.54 ± 0.08^a^	1.81 ± 0.07^b^	2.41 ± 0.23^a^	2.47 ± 0.10^a^	< 0.001
SR (%)	100.00 ± 0.00	100.00 ± 0.00	93.33 ± 11.55	98.33 ± 2.89	0.51

In the same study (Viagem et al. 2026), we reported that the apparent digestibility coefficients (ADC) differed significantly among diets (*p* < 0.05) (Table 3). The FM diet showed the highest digestibility of dry matter, fat, and energy. The SB diet exhibited protein digestibility comparable to that of FM and demonstrated relatively high overall nutrient utilization. In contrast, the SW diet showed significantly lower digestibility of dry matter, fat, and energy, although it had the highest protein digestibility among treatments. Carbohydrate digestibility was slightly higher in the FM diet than in the SB and SW diets.

**Table 3:** Apparent digestibility coefficient of nutrients in the experimental diets.

Nutrients	Treatments			*p* value
	FM	SB	SW	
Dry matter (%)	92.0 ± 0.4^a^	89.5 ± 0.4^b^	71.3 ± 0.3^c^	< 0.001
Protein (%)	80.7 ± 0.7^b^	81.1 ± 0.3^b^	84.5 ± 0.3^a^	< 0.001
Carbohydrate (%)	68.7 ± 0.1^a^	65.1 ± 0.8^b^	64.1 ± 0.4^b^	< 0.001
Fat (%)	94.8 ± 0.8^a^	92.3 ± 0.1^b^	55.0 ± 0.9^c^	< 0.001
Energy (%)	86.4 ± 0.2^a^	78.8 ± 0.7^b^	60.9 ± 0.6^c^	< 0.001

*Note:* The data are presented as the mean ± standard error (SE), and the parameters in the same row that share the same letter are not significantly different (*p* > 0.05). Different superscript letters within the same row indicate significant differences among dietary treatments (*p* < 0.05). Feed intake and feed conversion ratio could not be determined for the NM group, because these fish received no formulated feed.

Abbreviations: CF: condition factor; FBW: final body weight; FCR: feed conversion ratio; FM: fishmeal; FI: feed intake; IBW: initial body weight; NM: Natural meal; SB: soybean; SGR: specific growth rate; SR: survival rate; SW: seaweed (*Kappaphycus* sp.).

### 3.2 Intestinal Microbial Diversity

The composition of the microbiota community indicated that dietary treatments had significant effects on community structure, demonstrating diet‐specific responses that influence community assembly. In the DCA plot (Figure 1A), Axis 1 and Axis 2 explained 46.2% and 20.1% of the variance, respectively, showing a clear separation of the *Kappaphycus* sp. (SW) treatment from the others along Axis 1, while the natural meal (NM), soybean (SB), and fishmeal (FM) groups exhibited partial overlap. The Multidimensional Scaling (MDS) plot (Figure 1B) corroborated these findings, showing distinct clustering of dietary treatments consistent with differences in microbial community composition.

**Figure 1:**
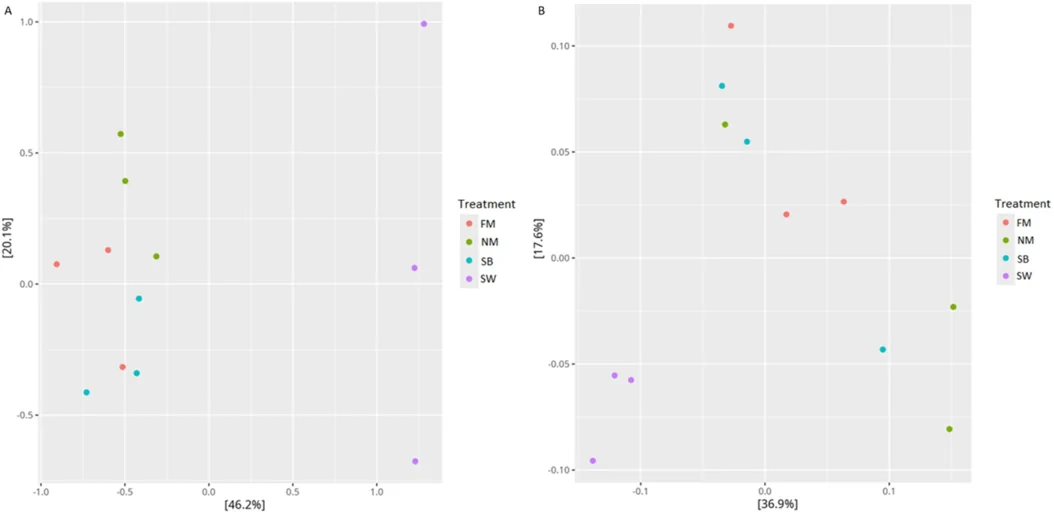
The Detrended correspondence analysis (A) and Multidimensional Scaling ordination (B) plots illustrate the beta diversity among treatments based on community composition in Nile tilapia. FM: fishmeal diet; NM: natural meal; SW: represents a sample of tilapia fed a diet containing *Kappaphycus* sp.; SB: soybean‐based diet.

Dietary treatment significantly influenced intestinal bacterial community composition (PERMANOVA, *F* = 4.053, *R* ^2^ = 0.603, *p* = 0.001; 4999 permutations) (Table S1). The multivariate dispersion did not differ significantly among treatments (PERMDISP, *F* = 0.321, *p* = 0.797), indicating similar within‐group dispersion across dietary treatments (Table S2).

The alpha diversity indices revealed that dietary treatments significantly affected the gut microbiota diversity of Nile tilapia (*p* < 0.05). Both the Shannon (Figure 2A) and Simpson (Figure 2B) indices were highest in fish fed the *Kappaphycus* sp. (SW) diet and lowest in fish receiving the natural meal (NM) diet, with the soybean meal (SB) and fishmeal (FM) groups intermediate. In pairwise comparisons, only the difference between the SW and NM groups reached statistical significance; the SB and FM groups did not differ from each other or from the remaining treatments.

**Figure 2:**
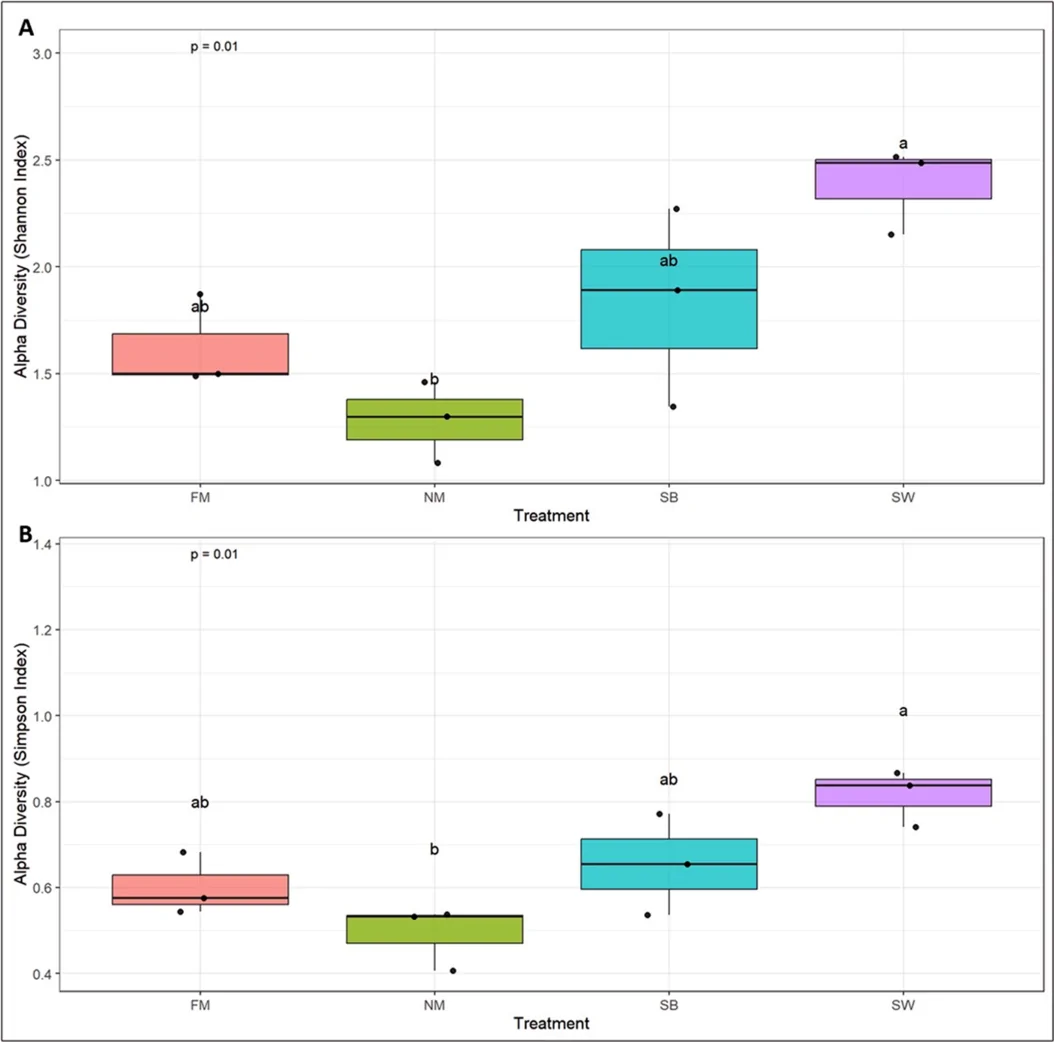
Alpha diversity indices of gut microbiota in Nile tilapia fed different experimental diets. (A) Shannon index and (B) Simpson index showing microbial diversity across treatments. FM: fishmeal‐based diet; NM: natural meal diet; SB: soybean meal‐based diet; and SW: diet containing *Kappaphycus* sp. Different letters above the boxes indicate significant differences among treatments (*p* < 0.05).

### 3.3 Intestinal Microbiota Composition

The phyla *Fusobacteriota* and *Pseudomonadota* dominated the microbial communities across all treatments, while variations in *Bacteroidota* were observed in fish fed a diet containing *Kappaphycus* sp. (SW) (Figure 3A). At the genus level (Figure 3B), *Cetobacterium* was the predominant taxon in all dietary treatments (NM: 70.03%; FM: 61.18%; SB: 56.42%; SW: 34.67%). However, notable differences were observed among the diets. In fish fed a diet containing *Kappaphycus* sp., *Arenimonas* (16.58%), *Dinghuibacter* (9.98%), and *Pseudomonas* (9.68%) were the most abundant genera, accompanied by marked increases in *Shewanella* (6.39%) and *Lactobacillus* (3.92%). The soybean‐based diet was dominated by *Shewanella* (7.75%), *Pseudomonas* (5.08%), and *Crenobacter* (5.07%). In the fishmeal‐based diet, *Fuscovulum* (8.29%) and *Bacillus* (4.38%) were the most dominant genera, whereas in the NM group, *Fuscovulum* (6.75%) and *Crenobacter* (4.56%) were the most abundant.

**Figure 3:**
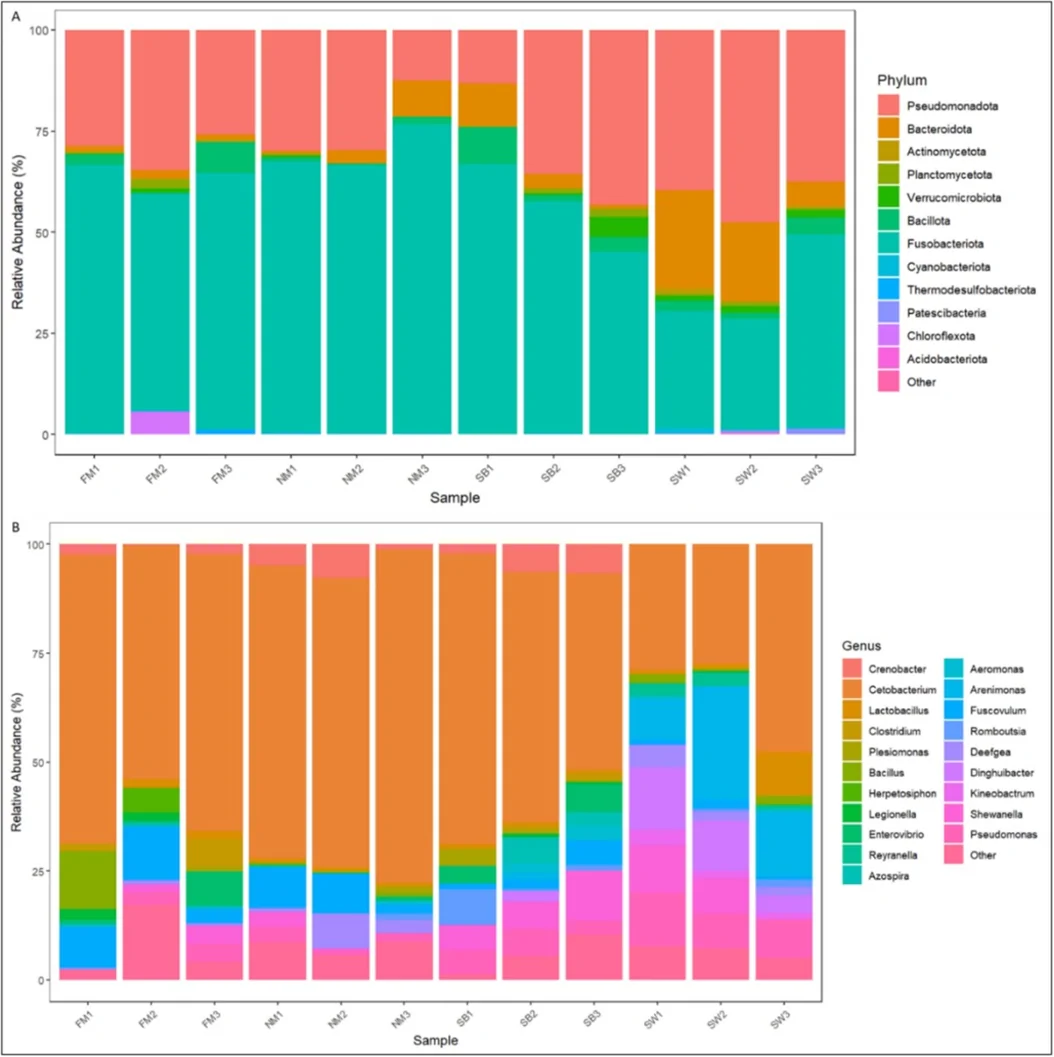
Relative abundance of the gut microbiota community at the Phylum (A) and genus (B) levels for Nile tilapia (*Oreochromis niloticus*) fed fishmeal (FM), soybean (SB), diet containing *Kappaphycus* sp. (SW), and natural meal (NM). Others represent the genera and Phylum with an abundance of less than 1% in each dietary treatment.

### 3.4 Differential Abundance

Differential abundance analysis revealed that under the soybean‐based (SB) diet, the relative abundance of the genera *Romboutsia*, *Turicibacter*, *Dinghuibacter*, *Kineobactrum*, *Cloacibacterium*, and *Propionivibrio* increased significantly, whereas Aquihabitans, Bacillus, Ralstonia, and Veillonella decreased compared with the fishmeal (FM) diet (Figure S1). When compared with the natural meal (NM) diet, the SB diet was associated with higher abundances of *Dinghuibacter*, *Terrimicrobium*, *Kineobactrum*, *Chthonobacter*, *Gemmata*, *Corynebacterium*, *Niveibacterium*, *Clostridium*, *Cloacibacterium*, *Propionivibrio*, *IMCC26207*, and *Azospira*, and lower abundances of *Roseomonas*, *Veillonella*, *Bacillus*, and *Deefgea* (Figure S2).

The diet containing *Kappaphycus* sp. (SW) led to increases in *Romboutsia*, *Dinghuibacter*, *Kineobactrum*, *Roseomonas*, *Flavobacterium*, *Pseudorhodoplanes*, *Bauldia*, *Mariniblastus*, and *Pirellula* compared with the FM diet. Conversely, the abundances of *Nordella*, *Rubrivivax*, *Ralstonia*, *Niveibacterium*, *Azospira*, *Dechloromonas*, *Tundrisphaera*, *Crenobacter*, *Veillonella*, *Terrimicrobium*, *Methylocystis*, *Enterovibrio*, *Plesiomonas*, *Pseudaeromonas*, *Anaerococcus*, and *Paenibacillus* were reduced (Figure S3). When compared with the NM diet, the SW treatment increased the abundance of *Dinghuibacter*, *Kineobactrum*, *Gemmata*, *Corynebacterium*, *Chthonobacter*, *Aquihabitans*, *Clostridium*, *Flavobacterium*, *Pseudorhodoplanes*, *Bauldia*, and *Mariniblastus*, while decreasing *Turicibacter*, *Nordella*, *Veillonella*, *Tundrisphaera*, *Rubrivivax*, *Dechloromonas*, *Methylocystis*, *Azospira*, *Plesiomonas*, *Enterovibrio*, *Crenobacter*, and *Pseudaeromonas* (Figure S4). These findings demonstrate that both the soybean and *Kappaphycus* sp. diets markedly altered the intestinal bacterial community structure of Nile tilapia, although several dominant genera remained unaffected.

The Venn diagram (Figure 4) illustrates that each diet supports distinct bacterial genera, with the greatest number of unique taxa observed in the diet containing *Kappaphycus* sp. (30; 23.6%), followed by FM (21; 16.5%), SB (8; 6.3%), and NM (7; 5.5%). Seventeen taxa (13.4%) were shared across all dietary treatments, representing a core microbiota, while several taxa were shared among specific diet combinations.

**Figure 4:**
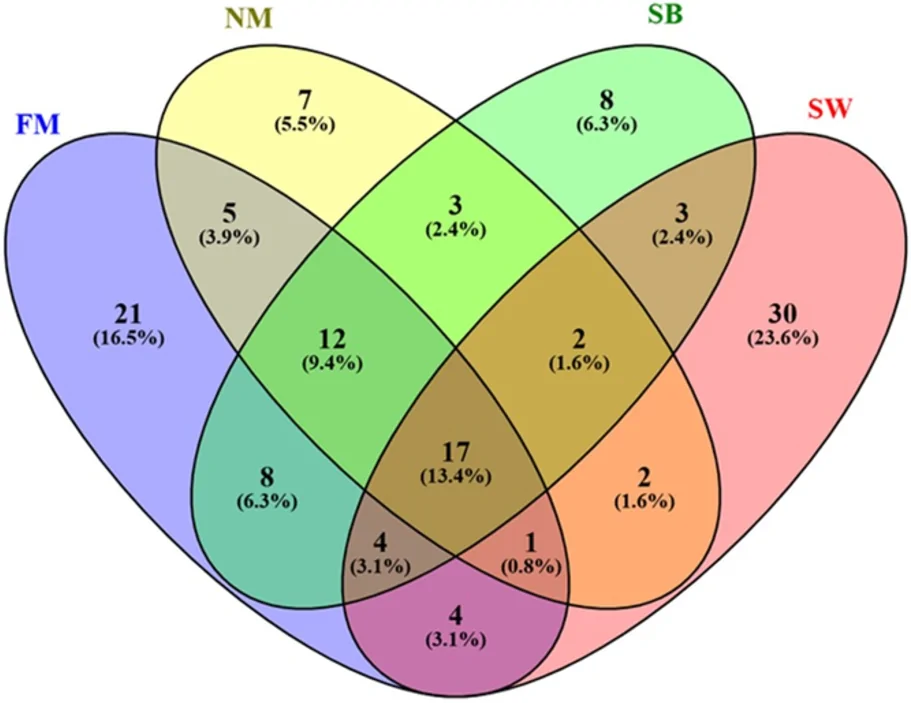
The Venn diagram shows the shared and unique gut microbiota of Nile tilapia fed with four diets: fishmeal (FM), soybean (SB), *Kappaphycus* sp. (SW), and natural meal (NM).

### 3.5 Predicted Functional Profiles

PICRUSt2‐based functional inference indicated diet‐related differences in predicted MetaCyc pathway abundances (Figure 5A). Fish fed a diet containing *Kappaphycus* sp. (SW) showed higher predicted abundances of pathways related to amino acid biosynthesis, including the superpathway of l‐isoleucine biosynthesis (PWY‐3001), l‐valine biosynthesis (VALSYN‐PWY), and l‐lysine biosynthesis III (PWY‐2942). The glucose‐1‐phosphate degradation pathway (GLUCOSE1PMETAB‐PWY) was also more represented in this diet.

**Figure 5:**
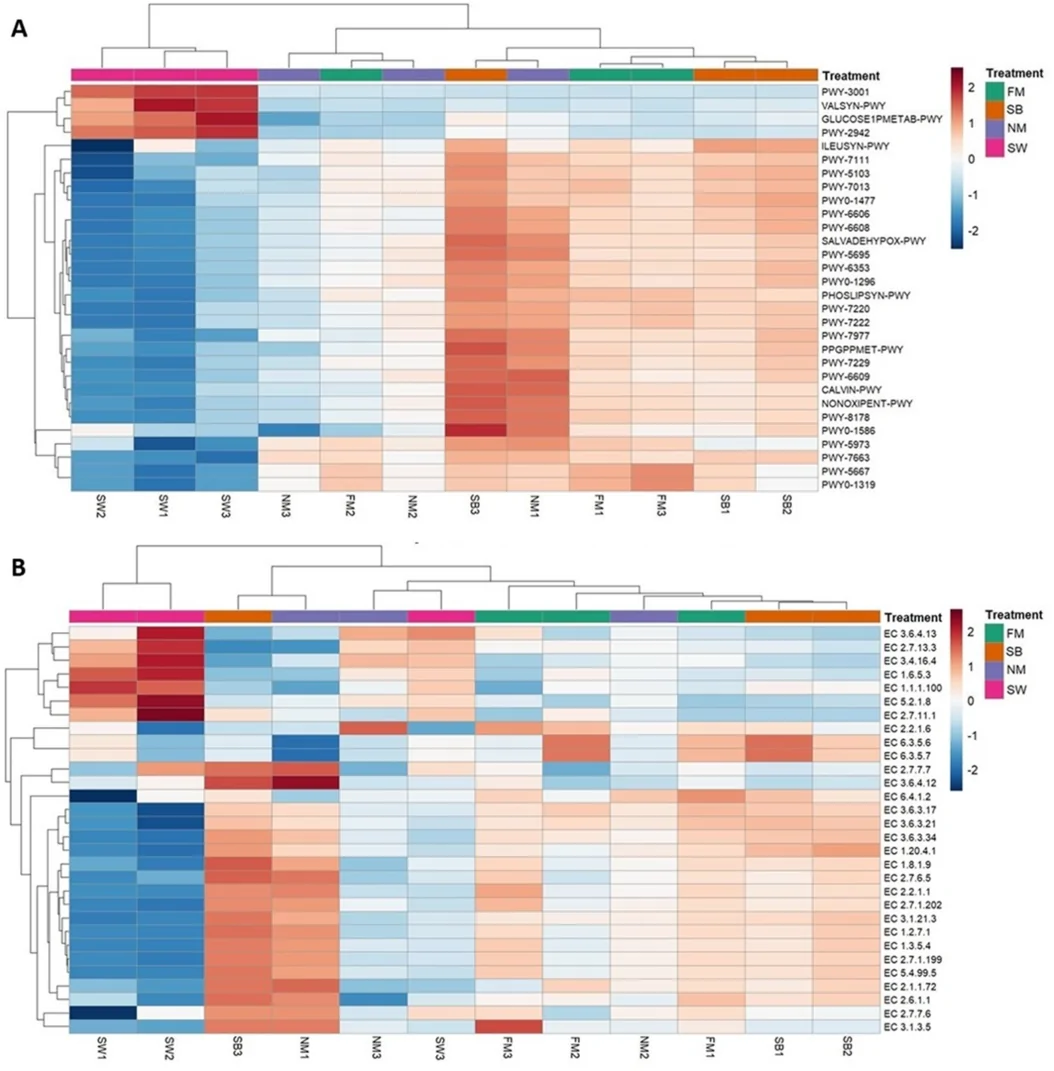
The hierarchical clustering of predicted metabolic pathways (A) and enzyme activities (B) across four dietary treatments: fishmeal (FM), natural meal (NM), soybean meal (SB), and a diet containing *Kappaphycus* sp. (SW). Red indicates higher predicted abundance, and blue indicates lower predicted abundance.

The SB diet showed a broader range of predicted functional categories, including central carbon metabolism, nucleotide degradation, de novo biosynthesis, and ppGpp metabolism (PPGPPMET‐PWY). Among the more represented pathways were guanosine nucleotides degradation III (PWY‐6608), adenosine nucleotides degradation II (SALVADHYPOX‐PWY), inosine 5′‐phosphate degradation (PWY‐5695), purine nucleotides degradation II (aerobic) (PWY‐6353), adenosine deoxyribonucleotides de novo biosynthesis II (PWY‐7220), and guanosine deoxyribonucleotides de novo biosynthesis II (PWY‐7222). Pathways associated with carbohydrate metabolism, including the Calvin–Benson–Bassham cycle (CALVIN‐PWY) and the non‐oxidative pentose phosphate pathway (NONOXIPENT‐PWY; PWY‐8178), were also more represented. Additionally, lipid biosynthesis pathways such as cis‐vaccenate biosynthesis (PWY‐5973) and anaerobic gondoate biosynthesis (PWY‐7663) showed higher predicted abundances.

The FM diet showed a similar pattern, including representation of nucleotide metabolism and the non‐oxidative pentose phosphate pathway (NONOXIPENT‐PWY), although at lower predicted abundances than SB. In contrast, the NM diet showed a relatively stable functional profile, with no pronounced differences in representation of specific MetaCyc pathways.

Predicted enzyme abundances (Figure 5B) also differed among diets. The SW diet showed higher predicted abundances of enzymes associated with transcriptional regulation, signal transduction, and fatty acid metabolism, including RNA helicase (EC 3.6.4.13), histidine kinase (EC 2.7.13.3), serine‐type d‐Ala‐d‐Ala carboxypeptidase (EC 3.4.16.4), and 3‐oxoacyl‐[acyl‐carrier‐protein] reductase (EC 1.1.1.100). The SB diet was associated with enzymes involved in redox processes (arsenate reductase [EC 1.20.4.1], thioredoxin‐disulfide reductase [EC 1.8.1.9]), amino acid metabolism (aspartate transaminase [EC 2.6.1.1]), tRNA charging (EC 6.3.5.6; EC 6.3.5.7), nucleotide processing (type I site‐specific deoxyribonuclease [EC 3.1.21.3]), and ATP‐binding transport systems (EC 3.6.3.17; EC 3.6.3.21; EC 3.6.3.34). The FM diet showed differences in enzymes involved in central carbon and nucleotide metabolism, whereas NM showed a comparatively narrower predicted profile.

## 4 Discussion

The intestinal microbiota plays a pivotal role in the health, nutrition, and disease resistance of Nile tilapia (*Oreochromis niloticus*). It influences host metabolism, immune function, and nutrient absorption (Huavas et al. 2024; Martinez‐Porchas et al. 2023). In this study, we investigated how replacing fishmeal with soybean meal or red seaweed (*Kappaphycus* sp.) affects the composition, diversity, and predicted functional potential of the tilapia gut microbiota. The results highlight the diet‐dependent plasticity of the Nile tilapia gut microbiota. This suggests that specific microbial taxa respond to dietary substrates and may contribute to carbohydrate metabolism and protein utilization. These findings highlight the potential of targeted dietary interventions to modulate the intestinal microbiota and support fish health and sustainable aquaculture.

### 4.1 Diet as a Driver of Gut Microbiota Composition

Gut microbiota analyses revealed that dietary treatments significantly influenced microbial community composition. This finding is consistent with previous studies showing that diet is a primary driver of the tilapia gut microbiota. Parata et al. (2020) reported that different diets markedly affected gut microbial composition and nutrient absorption in genetically improved farmed tilapia. Wang et al. (2022) similarly reported substantial effects of dietary treatments on the intestinal microbiota during early developmental stages.

Dietary protein has also been shown to influence microbiota structure. For example, do Vale Pereira et al. (2024) reported that dietary protein quality is critical for maintaining intestinal mucosal integrity and regulating the growth of luminal microbes. Similarly, Yang et al. (2021) demonstrated that different dietary protein levels can alter gut microbiota composition and community balance in tilapia. Our results extend these findings by showing that both plant‐ and seaweed‐based protein sources can modulate gut microbiota without disrupting its core structure.

### 4.2 Effects of a Diet Containing Soybean and *Kappaphycus* Sp. on Microbial Diversity

Dietary composition also significantly shapes gut microbial diversity. The alpha‐diversity patterns observed in this study align with growing evidence that alternative feed ingredients can enhance both taxonomic richness and evenness in the Nile tilapia gut microbiota. Seaweeds such as *Kappaphycus* sp. contain complex polysaccharides, bioactive metabolites, and micronutrients, which serve as fermentable substrates and modulators of gut conditions. Inclusion of these compounds into aquafeeds typically promotes a broader and more evenly distributed microbial community (Guillén et al. 2022; Shannon et al. 2021; Siddik et al. 2023), which is consistent with the higher alpha diversity recorded in tilapia fed the diet containing *Kappaphycus* sp. in the present study. Consistent with previous reports, seaweed‐based ingredients appear to function as prebiotic‐like components that support microbial diversity and host physiology in aquaculture species (Siddik et al. 2023; Siddik et al. 2024).

In contrast, no significant differences in alpha diversity were observed between fish fed soybean‐based and fishmeal‐based diets. Although soybean‐fed tilapia displayed slightly higher diversity, these differences were modest, suggesting that fishmeal replacement with soybean meal under the present conditions did not substantially alter overall microbial richness or evenness. This observation aligns with reports showing that properly processed soybean formulations can sustain or modestly enhance microbiota diversity by supplying fermentable carbohydrates or modifying protein availability (Leeper et al. 2023). Enzymatic or fermentative treatments of soybean meal, in particular, have been shown to increase microbial diversity in tilapia (Wang et al. 2023). Conversely, fishmeal diets, while highly digestible and supportive of growth, may favor a narrower range of proteolytic taxa, resulting in adequate but not maximal microbial diversity.

The relatively low diversity and distinct microbial profile observed in the natural meal (NM) group likely reflect nutritional heterogeneity and limited substrate availability rather than a targeted dietary formulation effect. Diets that are low in protein or nutritionally imbalanced have been associated with reduced microbial diversity, potentially compromising microbial resilience and host metabolic flexibility, and increasing susceptibility to opportunistic pathogens (Zhu et al. 2020). However, it should be noted that some studies, such as Yang et al. (2021) have reported stable alpha diversity across protein levels despite pronounced shifts in microbial composition, highlighting the complexity of diet–microbiota interactions.

### 4.3 Core Microbiota Stability and Diet‐Responsive Taxa

Across all dietary treatments, the gut microbiota of Nile tilapia was dominated by the phyla *Fusobacteriota* and *Pseudomonadota*, with an increased relative abundance of *Bacteroidota* in fish fed the *Kappaphycus* diet. This pattern is consistent with meta‐analyses identifying *Proteobacteria* and *Fusobacteria* as major constituents of the tilapia core microbiota, along with *Actinobacteria* and *Firmicutes* (Martinez‐Porchas et al. 2023). The persistence of *Fusobacteriota* and *Pseudomonadota* across diets indicates a stable core microbiota, while diet‐induced fluctuations in minor phyla, such as *Bacteroidota*, reflect selective modulation by dietary components.

At the genus level, *Cetobacterium* dominated across all treatments, consistent with its identification as a core genus in freshwater fish, particularly tilapia (Siddik et al. 2024; Wang et al. 2022; Younes et al. 2023). *Cetobacterium* plays a key role in peptide and amino acid fermentation and vitamin B12 production, supporting host nutrition and metabolism (Qi et al. 2023; Tsuchiya et al. 2008). Its dominance across diets underscores its importance as a stable mutualistic component of the tilapia gut ecosystem. Beyond *Cetobacterium*, dietary inclusion of soybean and *Kappaphycus* sp. led to notable shifts in microbial composition. Increases in taxa such as *Pseudomonas* and *Shewanella* may reflect responses to polysaccharides or other dietary substrates. The enrichment of *Lactobacillus* in the *Kappaphycus* diet suggests a prebiotic effect, likely due to fermentable fibers or bioactive compounds that selectively promote lactic acid bacteria (Cherry et al. 2019; Lopez‐Santamarina et al. 2020). Such changes may enhance short‐chain fatty acid (SCFA) production and contribute to improved gut ecological balance and host health, although SCFA concentrations were not measured in this study.

### 4.4 Functional Implications of Diet‐Driven Microbial Shifts

PICRUSt2‐based functional predictions suggested that dietary composition was associated with distinct predicted metabolic profiles within the intestinal microbiota in Nile tilapia. Because these predictions are inferred from 16S rRNA gene sequences rather than direct measurements of gene expression or metabolite production, they should be interpreted as hypotheses regarding the functional potential of the microbial communities rather than evidence of active metabolic processes. Nevertheless, the predicted pathway enrichment broadly paralleled the observed differences in growth performance and nutrient digestibility, providing biologically plausible hypotheses that warrant confirmation using metagenomic, metatranscriptomic, or metabolomic approaches.

Fish fed a diet containing *Kappaphycus* sp. (SW) showed selective enrichment of predicted amino acid biosynthesis pathways, particularly the superpathway of l‐isoleucine biosynthesis (PWY‐3001), l‐valine biosynthesis (VALSYN‐PWY), and l‐lysine biosynthesis III (PWY‐2942). Concurrently, the glucose‐1‐phosphate degradation pathway (GLUCOSE1PMETAB‐PWY) was elevated, suggesting enhanced carbohydrate turnover. The diet containing *Kappaphycus* sp. also enriched microbial genera such as *Flavobacterium* and *Bauldia*, which are associated with polysaccharide degradation and nutrient recycling (Kappelmann et al. 2019; Shannon et al. 2022). This functional profile aligns with the significantly higher protein digestibility observed in the SW group (84.5%) and supports the numerically improved feed conversion ratio (FCR: 0.90). A greater predicted microbial capacity for amino acid synthesis and carbohydrate utilization may therefore have contributed to efficient protein assimilation and competitive growth performance (FBW: 37.71 g; SGR: 2.13%).

The SB diet showed the most pronounced differences in predicted microbial functional profiles. Several nucleotide degradation pathways were enriched, including guanosine nucleotides degradation III (PWY‐6608), adenosine nucleotides degradation II (SALVADHYPOX‐PWY), inosine 5′‐phosphate degradation (PWY‐5695), and purine nucleotides degradation II (aerobic) (PWY‐6353). At the same time, pathways involved in de novo nucleotide biosynthesis, including adenosine deoxyribonucleotides de novo biosynthesis II (PWY‐7220) and guanosine deoxyribonucleotides de novo biosynthesis II (PWY‐7222), were also enriched, suggesting elevated nucleotide turnover and cellular proliferation potential. Enrichment of ppGpp metabolism (PPGPPMET‐PWY) further suggests enhanced microbial stress adaptation and regulatory responsiveness.

The SB diet was also associated with enrichment of pathways related to central carbon metabolism, including the Calvin–Benson–Bassham cycle (CALVIN‐PWY) and the non‐oxidative pentose phosphate pathway (NONOXIPENT‐PWY; PWY‐8178). Lipid‐associated pathways, such as cis‐vaccenate biosynthesis (PWY‐5973) and anaerobic gondoate biosynthesis (PWY‐7663), were also enriched. Genera enriched under the SB diet, including *Romboutsia* and *Turicibacter*, are linked to amino acid and lipid metabolism (Lynch et al. 2023; Yin et al. 2023). Collectively, these differences in predicted microbial functions occurred alongside the higher growth performance observed in SB‐fed fish (FBW: 39.42 g; WG: 29.37 g; SGR: 2.20%). These findings may indicate a relationship between changes in microbial functional profiles and nutrient utilization, although further investigation is needed to establish such associations.

The FM diet shared several functional features with SB, particularly enrichment of nucleotide metabolism and the non‐oxidative pentose phosphate pathway (NONOXIPENT‐PWY), although at comparatively lower predicted abundances. This functional profile was observed alongside higher growth performance (FBW: 38.32 g; SGR: 2.17%) and relatively high carbohydrate (68.7%) and fat (94.8%) digestibility coefficients. The enrichment of central carbon pathways may be associated with the efficient utilization of dietary nutrients. In contrast, the NM diet was associated with a comparatively stable and less specialized functional profile, without marked overrepresentation of key MetaCyc pathways. This pattern coincided with lower growth indices (FBW: 22.15 g; WG: 12.31 g; SGR: 1.31%) and a reduced condition factor, potentially reflecting differences in microbial metabolic activity and nutrient transformation among dietary treatments.

Predicted enzyme activity abundances followed similar trends at the pathway level. The SW diet was associated with higher predicted abundances of enzymes involved in transcriptional regulation and metabolic processing, including RNA helicase (EC 3.6.4.13), histidine kinase (EC 2.7.13.3), serine‐type d‐Ala‐d‐Ala carboxypeptidase (EC 3.4.16.4), and 3‐oxoacyl‐[acyl‐carrier‐protein] reductase (EC 1.1.1.100). These changes may reflect differences in regulatory processes, cell wall remodeling, and fatty acid metabolism. The observed enzymatic profiles were also associated with differences in protein digestibility and nutrient utilization.

The SB diet was associated with enrichment of enzymes involved in redox balance (arsenate reductase [EC 1.20.4.1], thioredoxin‐disulfide reductase [EC 1.8.1.9]), amino acid metabolism (aspartate transaminase [EC 2.6.1.1]), tRNA charging (EC 6.3.5.6; EC 6.3.5.7), nucleotide processing (type I site‐specific deoxyribonuclease [EC 3.1.21.3]), and ATP‐binding transport systems (EC 3.6.3.17; EC 3.6.3.21; EC 3.6.3.34). This pattern suggests increased representation of microbial functions related to biosynthesis, protein metabolism, and cellular transport, which may be associated with growth performance observed under the SB diet. The FM diet was associated with moderate enrichment of enzymes involved in central carbon and nucleotide metabolism, consistent with the high nutrient digestibility recorded for this diet. In contrast, the NM group was associated with fewer enriched enzymes, suggesting a comparatively lower representation of microbial metabolic functions and lower growth efficiency.

The predicted functional profiles suggest that diets associated with greater representation of pathways involved in amino acid biosynthesis, central carbon metabolism, nucleotide turnover, and lipid biosynthesis, particularly SB followed by FM and SW, were also associated with higher growth performance and nutrient utilization. In contrast, the relatively less variable functional profile observed in the NM group coincided with lower growth outcomes. These findings suggest that diet‐induced differences in the predicted metabolic potential of the gut microbiota may be related to differences in Nile tilapia growth and nutrient utilization. However, PICRUSt2 provides functional predictions based on 16S rRNA gene data rather than direct measurements of microbial gene expression or metabolite production. Therefore, these predictions should be interpreted with caution and may not fully reflect the metabolic activity of the gut microbiota under different dietary conditions. Future research incorporating metatranscriptomic or metaproteomic approaches could help validate these predictions and provide further insight into active microbial metabolic processes potentially involved in nutrient assimilation and growth performance. These findings should also be interpreted in the light of the modest level of replication (three tanks per treatment, each represented by a single pooled sample) and the single sampling point at the end of the feeding trial. In addition, several of the taxa identified as differentially abundant showed very large fold changes because they were absent or nearly absent from one of the treatment groups, and those contrasts are better regarded as qualitative than as quantitative estimates of effect size.

## 5 Conclusion

This study suggests that dietary protein source may influence gut microbiota composition and predicted metabolic potential in Nile tilapia. Under the conditions of this study, diets containing soybean meal and *Kappaphycus* sp. could replace fishmeal without compromising survival, while being associated with distinct microbial profiles linked to nutrient metabolism and growth efficiency. The soybean meal diet resulted in the highest growth performance and was associated with enrichment of pathways related to nucleotide biosynthesis, redox balance, and central carbon metabolism, which may reflect differences in microbial activity and nutrient utilization. In contrast, the *Kappaphycus* sp. diet increased microbial diversity and was associated with enrichment of amino acid biosynthesis and carbohydrate‐utilization pathways, alongside higher protein digestibility and similar growth with soybean and fishmeal diets. A relatively stable core microbiota dominated by *Cetobacterium* was maintained across diets, suggesting that some key microbial taxa may remain relatively stable despite dietary changes. The observed associations among predicted microbial functions, nutrient digestibility, and fish growth suggest that diet‐related changes in the gut microbiota may be linked to nutrient utilization and host performance. However, as microbial functions were predicted using PICRUSt2, further validation using metagenomic or metatranscriptomic approaches is needed to confirm these functional predictions and better understand their potential biological relevance.

## Author Contributions

**Leonildo dos Anjo Viagem:** conceptualization, investigation, funding acquisition, writing – original draft, methodology, writing – review and editing, visualization, formal analysis, data curation. **Jean Nepomuscene Hakizimana:** methodology, validation, writing – review and editing, visualization. **Cyrus Rumisha:** methodology, validation, visualization, writing – review and editing; supervision. **Brunno da Silva Cerozi:** methodology, validation, visualization, writing – review and editing, supervision. **Gerald Misinzo:** methodology, validation, visualization, writing – review and editing, supervision.

## Ethics Statement

The protocol and procedures employed in this study were reviewed and approved by the Ethics Committee on the Use of Animals at the College of Agriculture, University of São Paulo, Brazil (Protocol No. CEUA 3151270525).

## Conflicts of Interest

The authors declare no conflicts of interest.

## Supporting information


**Figure S1.** Volcano plot showing differential abundance of intestinal bacterial taxa in Nile tilapia fed soybean meal (SB) versus fishmeal (FM) diets. Labeled taxa indicate those with the strongest and most significant responses to dietary treatment. Up represents the taxa with significant increases in abundance in the SB diet; Down represents those significantly decreased, and NS represents non‐significant taxa.

**Figure S2.** Volcano plot showing differential abundance of intestinal bacterial taxa in Nile tilapia fed soybean meal (SB) versus natural meal (NM) diets. Labeled taxa indicate those with the strongest and most significant responses to dietary treatment. Up represents the taxa with significant increases in abundance in the SB diet; Down represents those significantly decreased, and NS represents non‐significant taxa.

**Figure S3.** Volcano plot showing differential abundance of intestinal bacterial taxa in Nile tilapia fed *Kappaphycus* sp. (SW) versus fishmeal (FM) diets. Labeled taxa indicate those with the strongest and most significant responses to dietary treatment. Up represents the taxa with significant increases in abundance in the SW diet; Down represents those significantly decreased, and NS represents non‐significant taxa.

**Figure S4.** Volcano plot showing differential abundance of intestinal bacterial taxa in Nile tilapia fed *Kappaphycus* sp. (SW) versus natural meal (NM) diets. Labeled taxa indicate those with the strongest and most significant responses to dietary treatment. Up represents the taxa with significant increases in abundance in the SW diet; Down represents those significantly decreased, and NS represents non‐significant taxa.

**Table S1.** PERMANOVA results for the effect of dietary treatment on bacterial community composition. PERMANOVA was performed using Bray–Curtis dissimilarities calculated from Hellinger‐transformed bacterial abundance data, with 4,999 unrestricted permutations. R^2^ represents the proportion of variation in bacterial community composition explained by dietary treatment, Df (degrees of freedom), F (F‐statistic), and p‐value (significance). **Table S2.** Test of homogeneity of multivariate dispersion among dietary treatments. Homogeneity of multivariate dispersion was assessed using PERMDISP with 4,999 unrestricted permutations. The non‐significant result indicates that multivariate dispersion did not differ significantly among dietary treatments.

## Data Availability

The raw 16S rRNA gene sequence reads generated in this study have been deposited in the NCBI Sequence Read Archive under BioProject accession PRJNA1514724. All other datasets are available from the corresponding author upon reasonable request.
