# Supplementary material for: Dietary Soybean and Kappaphycus sp. Differentially Modulate Gut Microbiota Diversity and Predicted Functional Profile in Nile Tilapia (Oreochromis niloticus)
Source: Microbiologyopen. 2026 Oct 18;15(5):e70442. doi: 10.1002/mbo3.70442 (PMC13678284; doi:10.1002/mbo3.70442)
Supplement: Supplementary file 1 [file MBO3-15-e70442-s003.docx]

**Title: Dietary Soybean and *Kappaphycus* sp. Differentially Modulate Gut Microbiome Diversity and Predicted Functional Profile in Nile Tilapia (*Oreochromis niloticus*)**

**Journal name**: MicrobiologyOpen

**Authors**: Leonildo dos Anjo Viagem^1,2,3*^; Jean Nepomuscene Hakizimana^2^; Cyrus Rumisha^1^; Brunno da Silva Cerozi^4^ and Gerald Misinzo^2,5^

**Affiliation**

^1^Department of Animal, Aquaculture and Range Sciences, College of Agriculture, Sokoine University of Agriculture, Morogoro, Tanzania;

^2^SACIDS Africa Centre of Excellence for Infectious Diseases, SACIDS Foundation for One Health, Sokoine University of Agriculture, Morogoro, Tanzania;

^3^Institute of Natural Resources and Environment, Rovuma University, Cabo Delgado, Mozambique;

^4^Department of Animal Science, College of Agriculture, University of São Paulo, São Paulo, Brazil

^5^Department of Microbiology, Parasitology and Biotechnology, College of Veterinary Medicine and Biomedical Sciences, Sokoine University of Agriculture, Morogoro, Tanzania;

***Corresponding author**

Leonildo dos Anjo Viagem ([lviagem@unirovuma.ac.mz](mailto:lviagem@unirovuma.ac.mz))

**Table S1.** PERMANOVA results for the effect of dietary treatment on bacterial community composition. PERMANOVA was performed using Bray–Curtis dissimilarities calculated from Hellinger-transformed bacterial abundance data, with 4,999 unrestricted permutations. R² represents the proportion of variation in bacterial community composition explained by dietary treatment, Df (degrees of freedom), F (F-statistic), and *p-*value (significance).

| **Source** | **df** | **Sum of Squares** | **R²** | **F** | ***p*-value** |
| --- | --- | --- | --- | --- | --- |
| **Dietary treatment** | 3 | 0.89794 | 0.603 | 4.053 | 0.001 |
| **Residuals** | 8 | 0.59082 | 0.397 | — | — |
| **Total** | 11 | 1.48876 | 1.000 | — | — |

**Table 2. PERMDISP analysis**

**Table S2.** Test of homogeneity of multivariate dispersion among dietary treatments. Homogeneity of multivariate dispersion was assessed using PERMDISP with 4,999 unrestricted permutations. The non-significant result indicates that multivariate dispersion did not differ significantly among dietary treatments.

| **Source** | **df** | **Sum of Squares** | **Mean Square** | **F** | ***p*-value** |
| --- | --- | --- | --- | --- | --- |
| Dietary treatment | 3 | 0.007122 | 0.002374 | 0.321 | 0.797 |
| Residuals | 8 | 0.059119 | 0.007390 | — | — |
